# The Magnitude of Contralateral Suppression of Otoacoustic Emissions Is Ear- and Age-Dependent

**DOI:** 10.3390/jcm12134553

**Published:** 2023-07-07

**Authors:** Hung Thai-Van, Evelyne Veuillet, Marie-Thérèse Le Normand, Maxime Damien, Charles-Alexandre Joly, Pierre Reynard

**Affiliations:** 1 Institut de l’Audition, Institut Pasteur, Inserm, 75012 Paris, France; evelyne.veuillet@chu-lyon.fr (E.V.); maxime.damien@chu-lyon.fr (M.D.); charles-alexandre.joly01@chu-lyon.fr (C.-A.J.);; 2 Service d’Audiologie et d’Explorations Oto-Neurologiques, Hôpital Edouard Herriot, Hospices Civils de Lyon, 69003 Lyon, France; 3 Faculty of Medicine, Université Claude Bernard Lyon 1, 69100 Villeurbanne, France; 4 Laboratoire Psychopathologie et Processus de Santé, URP 4057, Université Paris Cité, 92100 Boulogne Billancourt, France

**Keywords:** medial olivocochlear efferent, uncrossed fibers, transient evoked otoacoustic emissions, contralateral acoustic stimulation, equivalent attenuation, right ear, left ear, general linear model

## Abstract

The maturation of the uncrossed medial olivocochlear (UMOC) efferent remains poorly documented to date. The UMOC efferent system allows listeners to not only detect but also to process, recognize, and discriminate auditory stimuli. Its fibers can be explored non-invasively by recording the effect of contralateral acoustic stimulation (CAS), resulting in a decrease in the amplitude of transient evoked otoacoustic emissions (TEOAE). The objective of the present cross-sectional study was to investigate how the effectiveness of this system varies with age in healthy subjects aged 8 years to adulthood. For this purpose, 120 right-handed native French-speaking subjects (57 females and 63 males) were divided into five age groups of 24 subjects each: 8y–10y, 10y–11y6m, 11y6m–13y, 13y–17y, and ≥18y. TEOAE amplitudes with and without CAS were recorded. The equivalent attenuation (EA) was calculated, corresponding to the change in TEOAE amplitude equivalent to the effect generated by CAS. General linear models were performed to control for the effect of ear, sex, and age on EA. No sex effect was found. A stronger EA was consistently found regardless of age group in the right ear compared to the left. In contrast to the right ear, for which, on average, EA remained constant across age groups, an increasingly weaker TEOAE suppression effect with age was found in the left ear, reinforcing the asymmetrical functioning of the UMOC efferent system in favor of the right ear in adulthood. Further studies are needed to investigate the lateralization of the UMOC efferent system and its changes over time in cases of atypical or reversed cortical asymmetries, especially in subjects with specific learning disorders.

## 1. Introduction

The central auditory nervous system (CANS) consists of ascending and descending auditory pathways that are closely interdependent and act in an integrated manner. From the peripheral auditory receptor, afferent fibers carry information to the auditory cortex (AC) through the brainstem via, successively, the cochlear nuclei, the superior olivary complex (SOC), the lateral lemniscus, the inferior colliculus, and the medial geniculate body [1]. In turn, the AC is the source of multiple efferent projections [2,3] to each of the auditory nuclei [4]. A contingent of efferent fibers originates from the medial SOC and forms the “Uncrossed Medial OlivoCochlear (UMOC)” efferent system that projects to the ipsilateral cochlea reaching directly to the outer hair cells (OHCs) [5] (for a recent review, see [6]). In doing so, the SOC serves as a relay station for the corticofugal pathway that originates from pyramidal neurons in layers V/VI of the primary AC [7]. Therefore, the AC is able to control and modulate OHCs activity in a top-down manner [8].

The CANS undergoes maturational changes that allow children not only to detect but also to process, recognize, and discriminate auditory stimuli. Auditory evoked potentials (AEPs) have been extensively used to study the developmental time course of the auditory afferent pathway. It is well known that AEP peaks shorten and become sharper with age, suggesting an increase in neural efficiency [9,10,11,12]. However, having long assumed that the latency of early AEPs reaches adult values within the first two years of life [13], more recent research suggests a longer developmental time course, particularly for the auditory brainstem, which continues to mature during childhood [14] and even adolescence [15], leading to the notion of continuous maturational plasticity. Similarly, late AEPs have shown that changes in AC extend into late adolescence [16]. Maturation of the auditory thalamocortical connections occurs between two and five years of age, with the final stage of structural maturation of AC beginning later (at the earliest between 6 and 12 years) (see [17] for a detailed review). Such maturational changes underpin the development of increasingly complex auditory processing, allowing for a strong improvement in speech understanding, especially in noise, in late childhood and adolescence [18]. This is probably made possible not only by stronger cortical interaction within the same hemisphere but also by increasing communication between the two hemispheres. As cortical regions continue to mature in adolescence, functional changes may still occur in early adulthood [19].

In contrast, the maturation of the auditory corticofugal pathway remains much less documented. This is even more surprising as the UMOC efferent system can be explored non-invasively in clinical routine. One of the major advances in modern audiology is credited to Kemp [20,21], who first recorded, in the auditory canal, sounds produced by the contraction of OHCs in response to brief acoustic clicks and identified them as transient evoked otoacoustic emissions (TEOAEs). Due to the UMOC fibers being inhibitory, the addition of a contralateral acoustic stimulation (CAS) leads to a TEOAE amplitude decrease [22,23] called the “contralateral suppressive effect” (CSE). While recent studies in animals [24,25,26,27] provide strong anatomical and functional evidence for the existence of central control of OHCs by multiple corticofugal loops, in humans, such demonstrations have long remained indirect. For instance, in right-handers, the CSE has been reported to be stronger in the right ear, reflecting a rightward asymmetry in UMOC efferent system function [28,29], this asymmetry being absent in right-handed schizophrenic patients [30]. Surgical resection of the primary AC has been shown to suppress the CSE [31], whereas intracortical electrical stimulation of Heschl’s gyrus increases it [32]. A recent functional MRI study in healthy adult subjects has confirmed the existence of direct connections between auditory structures, including Heschl’s gyrus, the planum temporale, and the SOC [33].

From a developmental perspective, the CSE is present at birth [34] but is reduced in preterm neonates [35] with a greater suppressive effect on the right ear [36,37], which may reflect an early lateralization process. It has been reported that the magnitude of CSE slowly decreases during infancy until the age of 3 years [38]. A recent study found no effect of age on the amplitude of CSE in preschoolers aged 3–6 years [39]. Several studies have been conducted on older children, but to our knowledge, never on children over 14 years of age, and often on groups covering broad age ranges up to 4 years [40,41,42,43]. Data in the literature have reported differences in CSE amplitude between the right and left ear in children aged 9–10 years [44], 5–10 years [45], or 7–12 years [46]. Despite this, the fine-grained age-related changes in UMOC efferent system function during a highly plastic but vulnerable developmental period from childhood to early adulthood are still poorly understood.

As there are only a few studies on the effect of age on UMOC, and they are not conclusive, the aim of the present cross-sectional study was to examine how CSE varies with age in healthy subjects aged 8 to 35. Given that the cortical auditory areas are not fully mature until late adolescence and similar to the gradual maturation of the auditory afferent pathway, we expected to observe differences in CSE amplitude with age, but also depending on the test ear.

## 2. Materials and Methods

### 2.1. Participants

One hundred and twenty native French-speaking right-handed subjects, with a balanced number of females (*n* = 57) and males (*n* = 63) and a mean age of 13 ± 0.3 years (range 8–35), participated in the study. The experiment was carried out with the agreement of the Regional Ethics Committee (CPP Sud-Est IV Lyon, approval No. 09/086 for adults and 04/008 for children). Informed consent was obtained from all participants (all children with verbal assent). Handedness degree was assessed using the Edinburgh Handedness Inventory [47]. None of the participants had a history of communication, cognitive, neurological, attention, or autism spectrum disorders. The participating children performed average or above average academically, never had to repeat a grade, and had no specific learning disorders. None of them had undergone speech therapy, and all of them had a normal reading age, as measured by the “L’alouette” test [48]. Participants were divided into five age groups of 24 subjects each, as follows: 8y–10y (group 1), 10y–11y6m (group 2), 11y6m–13y (group 3), 13y–17 (group 4), and ≥18y (group 5).

The demographic characteristics by age group and sex and the laterality by age group are shown in Table 1.

All participants had normal otoscopy, air conduction hearing thresholds less than 15 dB HL at octave intervals from 0.25 to 8 kHz, and normal type A tympanograms (static acoustic admittance between 0.35 and 1.75 mmho and peak pressure between +50 to −100 daPa) in both ears. They also had ipsi- and contralateral acoustic reflex thresholds (ARTs) greater than 75 dB SPL in response to white noise bilaterally. TEOAEs were bilaterally present for all participants with an overall response amplitude of emissions exceeding the noise amplitude; that is, a signal-to-noise ratio (SNR) greater than 4 dB when measured with a non-linear click method at an intrameatal intensity of 81.5 ± 0.05 peak-equivalent (pe) SPL. The whole reproducibility level was greater than 50%, with stimulus stability exceeding 90%.

### 2.2. Apparatus

All measurements were performed in a soundproof booth. TEOAEs were recorded using the ILO92 system (Otodynamics Ltd., London, UK). The Otodynamics 5.6 clinical OAE software was used, and responses were recorded by a standard transducer (Otodynamics Model UGS TEOAE Probe). The probe, comprising a transmitter and a microphone, was fitted to the external auditory meatus with a foam rubber tip. CAS was delivered with a headphone (TDH49) using an AC40 clinical audiometer (Interacoustics, Middlefart, Denmark).

### 2.3. Procedure

The participants were instructed to sit quietly in a comfortable position and to keep silent. To avoid falling asleep or to keep them calm, a silent movie was played during this passive CSE measurement (cartoon for children and film for adults). Measurements were successively conducted on the right ear and left ear in random order.

TEOAEs were recorded in a linear mode using 80-µs clicks at a rate of 50/s, successively in the absence and presence of a 35 dBSL continuous noise (0.125–12 kHz with a flat spectrum) in the contralateral ear (minimal threshold = 0 dBHL—maximal threshold = 15 dBHL). The stimulus level was randomly adjusted to obtain 5 peak pressures between 60 and 72 peSPL (±1 dB) that were presented in 3 dB steps. CAS was manually switched on prior to the onset of TEOAE recordings. For each of the 5 ipsilateral stimulation levels, the first measurement was always carried out without CAS. The noise rejection level was set at 47.3 dB peSPL. Three hundred responses were averaged in a 3.2 to 20 ms-time window. Signal parameters were analyzed as global values (for the whole signal). This protocol provides an input/output function for the global TEOAE amplitude with and without CAS and calculates the equivalent attenuation (EA) according to the so-called Lyon procedure (see for more details [44,46,49]). As such, EA corresponds to the change (in dB) of the ipsilateral click-evoked OAEs equivalent to the effect generated by CAS. The more negative the EA, the stronger the TEOAE suppressive effect. By subtracting the EA measured on each ear (EA_right_–EA_left_), an asymmetry index (AI) can be obtained: the more negative it is, the stronger the CSE on the right ear.

### 2.4. Data Analysis

Two general linear models (GLMs), using JAMOVI version 1.6 (https://www.jamovi.org/, accessed on 20 June 2023), were run on three dependent variables (EA_right_, EA_left_, and AI) fitting the UMOC efferent system function. The first model used only one factor at a time: five age groups or sex. To ensure that the effect of age did not depend on our choice of age groups, we also investigated the relationships between age, as a continuous variable, and our three variables of interest (EA_right_, EA_left_, and AI) using the Spearman correlation test. A second model accounted for two factors: five age groups and sex with an ANOVA design. To account for multiple comparisons in the GLM analysis, all *p*-values were thresholded at a Bonferroni-corrected value of *p* = 0.5. Violin plots were drawn for data description, including a marker for the median value and a box for the interquartile range.

## 3. Results

**Figure 1:**
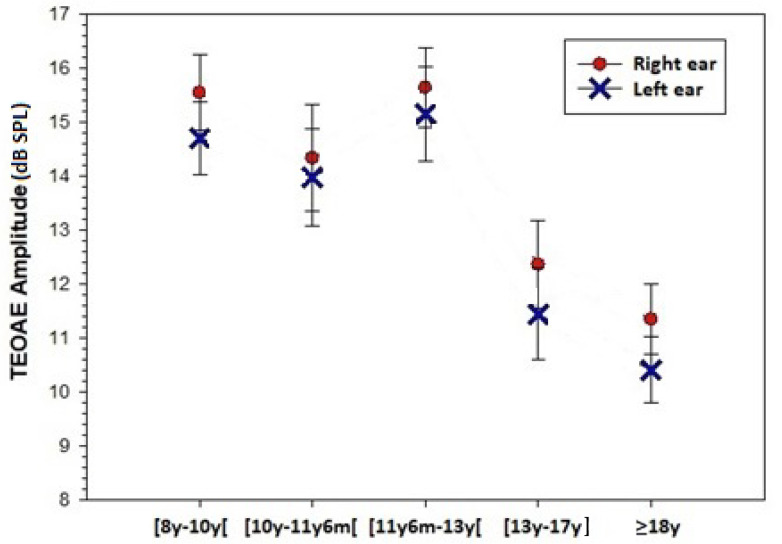
Average response amplitude of TEOAEs obtained for the 5 age groups on each ear. TEOAE: transient evoked otoacoustic emissions.

The average response amplitude of TEOAEs obtained for each age group on each ear is presented in Figure 1. The average values are lower on the LE than on the RE. A mixed-design ANOVA, with age as a fixed effect factor and the ear side as a random effects factor, revealed significant effects of the two factors: respectively, (F(4,119) = 7.287; *p* < 0.001) and (F(1, 119) = 9.085; *p* = 0.003), without significant interaction. However, none of the paired *t*-tests comparing TEOAE amplitude between the ears within each age group was significant.

**Figure 2:**
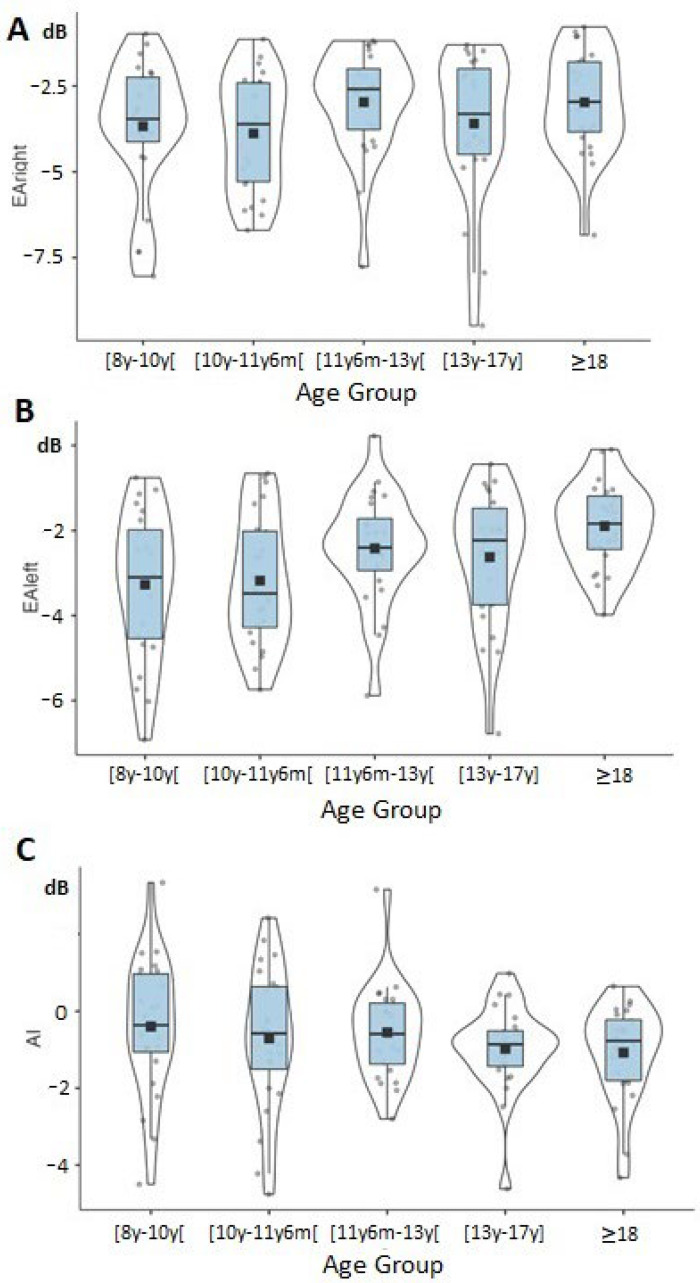
Data are plotted as violin plots showing right-ear equivalent attenuation (EA_right_) in panel (**A**), left-ear equivalent attenuation (EA_left_) in panel (**B**), and asymmetry index (AI) in panel (**C**) for each age group. Within each violin plot, the rectangular box represents the interquartile range (from bottom to top: lower quartile, upper quartile), the bold line the median, and the black square the mean value.

Figure 2 shows the CSE distribution among the five age groups. A significant age group effect was found for EA_left_ (F(4, 119) = 3.75, *p* = 0.007, η^2^ = 0.12) from 8 years to 13 years (β = 0.57, t(110) = 2.05, *p* = 0.04) and across the developmental trajectory from 8 years to adulthood (β = 0.57, t(110) = 3.32, *p* = 0.001). In contrast, no significant age group effect was found for EA_right_ (F(4, 119) =1.37 *p* = 0.25, η^2^ = 0.05) and AI (F(4, 119) = 0.90, *p* = 0.46, η^2^ = 0.03). The average EA and AI values for each ear are provided in Table 2 for each age group. Spearman correlation tests confirmed that age was statistically related to EA_left_ (rho = 0.27; *p*-value = 0.002) but not to EA_right_ (rho = 0.12; *p*-value = 0.2) or AI (rho = −0.14; *p*-value = 0.13).

**Figure 3:**
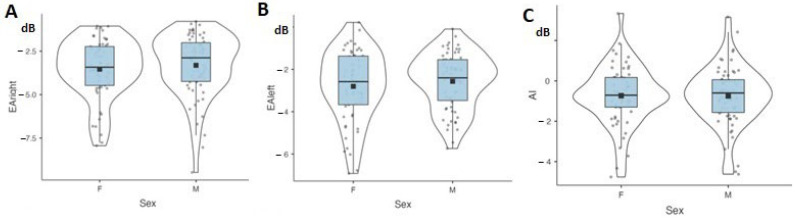
Data are plotted as violin plots showing right-ear equivalent attenuation (EA_right_) in panel (**A**), left-ear equivalent attenuation (EA_left_) in panel (**B**), and asymmetry index (AI) in panel (**C**) for male and female participants. Within each violin plot, the rectangular box represents the interquartile range (from bottom to top lower quartile, upper quartile), the bold line the median, and the black square the mean value.

With all ages combined, no significant sex effect was found for EA_right_ (F(1, 119) = 0.48, *p* = 0.40, η^2^ = 0.00), EA_left_ (F(1, 119) = 0.75, *p* = 0.39, η^2^ = 0.01), or AI (F(1, 119) = 0.00, *p* = 0.90, η^2^ = 0.00) (Figure 3). Average EA and AI values for each ear are provided in Table 3 for male and female patients (all ages combined).

**Figure 4:**
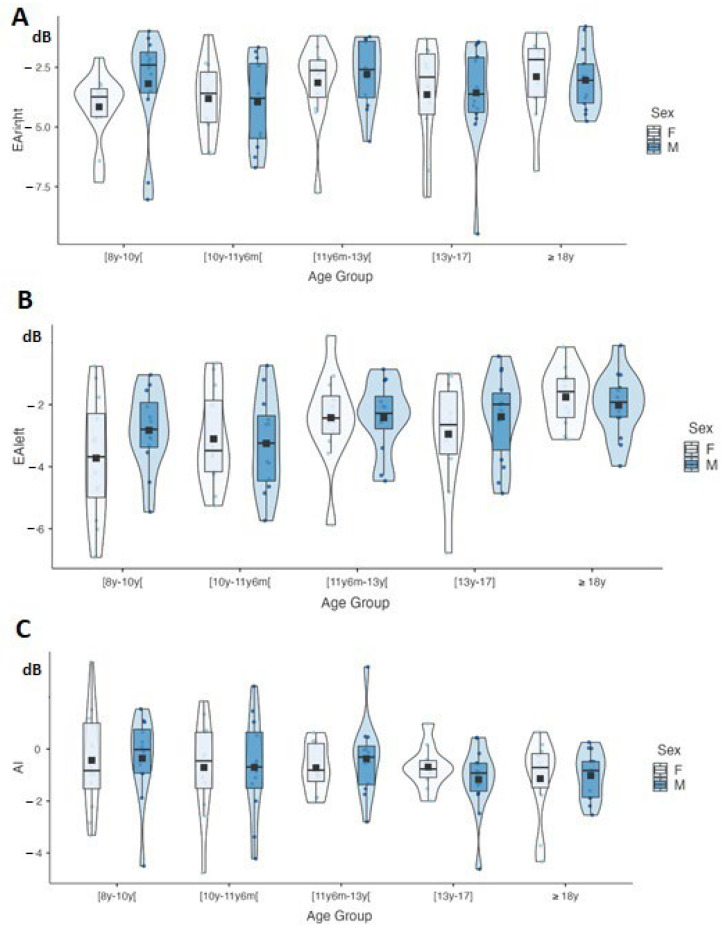
Data are plotted as violin plots showing right-ear equivalent attenuation (EA_right_) in panel (**A**), left-ear equivalent attenuation (EA_left_) in panel (**B**), and asymmetry index (AI) in panel (**C**). Within each violin plot, the rectangular box represents the interquartile range (from bottom to top lower quartile, upper quartile), the bold line the median, and the black square the mean value.

Finally, a two-way age group X sex factor test was performed to determine the influence of these two variables on CSE. Figure 4 shows the CSE distribution by age group and sex.

A significant age group effect was found for EA_left_ and AI (F(4, 119) = 9.85, *p* < 0.001, η^2^ = 0.26) from 10 years to 13 years (t(114) = 2.05, *p* = 0.04) and from 8 years to adulthood (t(114) = 3.32, *p* = 0.001). In contrast, there was no significant age group effect for EA_right_ (F(4, 119) = 1.64 *p* = 0.17, η^2^ = 0.06).

No significant sex effect was found for EA_left_ (F(1, 119) = 0.27, *p* = 0.60, η^2^ = 0.14) and EA_right_ (F(1, 119) = 0.20 *p* = 0.65, η^2^ = 0.04), nor for AI (F(1, 119) = 0.27, *p* = 0.60, η^2^ = 0.03).

No interaction for EA_right_ (F(4, 119) = 0.41, *p* = 0.80, η^2^ = 0.01), EA_left_ (F(4, 119) = 0.25, *p* = 0.90, η^2^ = 0.01), or AI (F(4, 119) = 0.25, *p* = 0.90, η^2^ = 0.01) was found.

Average EA and AI values for each ear are provided in Table 4, for male and female patients and each age group.

## 4. Discussion

In this study of typically developing right-handed subjects, we identified ear-dependent differences in the evolution of UMOC function between childhood and adulthood. As maturation of the auditory afferent pathway is gradual, we expected to observe differences in CSE amplitude with age, but also as a function of the ear. Unlike the right ear, for which, on average, EA remained constant across age groups, an increasingly weaker TEOAE suppression effect with age was found in the left ear. These results confirm those of previous studies observing that in adult right-handers, CSE is significantly stronger in the right ear [49]. This RE advantage in TEOAE suppression reinforces the concept of laterality of the UMOC function, which favors the action of the right olivocochlear tract over the left [50].

Here, no significant age group effect was found for EA on the right ear and AI. On the other hand, a significant age group effect was found for EA from age 8 to 13 and over the entire developmental trajectory from age 8 to adulthood for the left ear. Previous studies on groups with a wider age range revealed no significant asymmetry in a group of children aged 6 to 13 [51], while a mean laterality score significantly lower than 0, indicating right-ear predominance, was found in typically developing children aged 8 to 14 [42]. Notably, the present study is the only one to date to compare smaller age groups over a longer period (from childhood to adulthood), with a large and identical number of participants (N = 24) in each group. It underlines the fact that asymmetry of UMOC function may continue to develop well into childhood and adolescence.

Our results indicate that changes in the function of the UMOC efferent system with age are underpinned by a reduction in the CSE on the left ear, with stability on the right. This raises the question of what factors might explain this reduction in contralateral suppression, limited to the left ear. Firstly, one factor that may play a role is the presence or absence of TEOAEs of comparable amplitude between the two ears. Given that the CSE is based on the measurement of a change in TEOAE amplitude under the effect of acoustic stimulation, the functional integrity of the OHCs is paramount, and the presence of TEOAEs reflects this [21]. This was the case for all the participants in the present study since they all presented a global TEOAE response value of at least 4 dBpeSPL with more than 50% reproducibility in both ears. Strict selection criteria for tympanometry and audiometry were also used to exclude any potential peripheral effects on TEOAE amplitude. We observed a significant decrease in TEOAE amplitude with age, confirming previous studies [52,53]. This decrease was identical in both ears, with no difference in TEOAE amplitude between ears in each age group. Thus, if the TEOAE amplitude factor alone explained the decrease in contralateral suppression observed in the left ear, the latter should also have been observed in the right ear. Secondly, there is evidence that handedness may play a role in TEOAE amplitude and suppression [28]. However, this is unlikely to have been the case here since all participants were right-handed.

Rather, it may be asked whether the relative constancy of CSE as age increases can be explained by the completion of maturation processes. In a post-mortem histological and immunohistochemical study, Moore and Guan traced cortical maturation through the fetal period, infancy, childhood, and young adulthood [54]. Maturation of layers 4, 5, and 6 and the medial geniculate to the cortex was found to be complete by age 6. Later in childhood, maturation would take place in superficial layers IIIb, IIIa, and II of the cortex. By the age of 12, the density of mature axons has become equivalent to that of an adult [54]. The longer duration of axonal maturation could have implications for the emergence of auditory cortical functions [17,54]. Other central auditory processes undergo maturation during adolescence that continues into adulthood, including those associated with the predominance of right-ear listening in right-handed individuals [55,56]. It is possible that the asymmetrical changes in CSE observed here with age are linked to the maturation of more central processes, reinforcing the hypothesis of the existence of central control of OHCs function.

Finally, one may wonder why the UMOC efferent system function in the left ear does not reach stability as rapidly as in the right. This could be because the subcortical or cortical structures that control it develop later. However, descending loops are anatomically complex, and their function is still poorly understood. Our results suggest that, in right-handed children, UMOC fibers are involved in different maturation processes between the right and left ears. In the left ear (receiving uncrossed efferent fibers from the left superior olivary complex explored by a CAS passing through the right cochlear nucleus), this system would continue to mature by “calming down”. In the right ear (receiving uncrossed efferent fibers from the right superior olivary complex explored by a CAS passing through the left cochlear nucleus), the system could mature earlier as a reflection of the predominance of the left auditory cortex in right-handed subjects.

Here, we observed no statistically significant gender differences in TEOAE suppression and AI. Gender disparities in TEOAEs exist in both neonates and adults. Mac Faden et al. suggested that the degree of androgen exposure during prenatal development may be a contributing factor to these differences [57]. While women are known to have stronger click-induced OAEs than men [57], a study investigating contralateral TEOAE suppression as a function of gender showed that men had greater contralateral suppression, the exact reason for this difference being unclear [58]. Other studies have found no sex differences in the contralateral suppression of OAEs [59,60]. Among them, Jedrzejczak et al. measured TEOAE with and without CAS in 126 normal-hearing children aged 3–6 years. They found no significant effect of sex on TEOAE suppression [39,61].

Some questions remain: what is the exact starting point of this asymmetrical development, which in this study only manifests from the age of 11y6m? Previous research has shown protracted subcortical auditory maturation during adolescence, which may represent a “transitional point” between the enhanced response in childhood and the mature, albeit smaller, response in adulthood [15].

It is still possible that the samples studied here were too small; moreover, we observed a large inter-individual variability in adults but also in children in terms of CSE. In terms of learning acquisitions, all participants were described as having a “normal” reading age. Yet, each age group in the study covered a wide range of reading ages, which may have increased within-group variability.

Further studies are needed to study the maturation of UMOC function when cortical asymmetries are reversed or absent, as is the case in dyslexic subjects. If feasible, longitudinal studies would neutralize the inter-individual variability observed in the variables fitting the UMOC efferent system function.

## Figures and Tables

**Table 1:** Demographic characteristics.

		Group 1	Group 2	Group 3	Group 4	Group 5
Age (months)	Min	96	120	139	157	219
	Max	118	138	156	213	425
	Mean (SE)	108.3 (1.5)	130 (1.2)	147.2 (1.2)	181.3 (3.6)	279.5 (12.4)
Sex		12♀/12♂	12♀/12♂	12♀/12♂	10♀/14♂	11♀/13♂
Manual laterality (%)	Min	71.4	80	77.8	71.4	80
	Max	100	100	100	100	100
	Mean (SE)	91.5 (2.3)	95.1 (1.7)	93 (2.1)	92.9 (2.1)	97.3 (1.3)

♀: female subject; ♂: male subject.

**Table 2:** Average equivalent attenuation (EA) and asymmetry index (AI) values for each ear and age group.

Variables	Age Group	N	Mean	Lower	Upper	Median	SD	IQR
EA_right_	[8y–10y]	24	−3.67	−4.48	−2.86	−3.46	1.92	1.88
	[10y–11y6m]	24	−3.88	−4.59	−3.18	−3.61	1.67	2.88
	[11y6m–13y]	24	−2.97	−3.62	−2.31	−2.59	1.55	1.77
	[13y–17]	24	−3.60	−4.49	−2.70	−3.31	2.12	2.50
	≥18y	24	−2.97	−3.60	−2.35	−2.96	1.48	2.04
EA_left_	[8y–10y]	24	−3.27	−3.99	−2.56	−3.10	1.70	2.56
	[10y–11y6m]	24	−3.18	−3.82	−2.54	−3.48	1.51	2.26
	[11y6m–13y]	24	−2.42	−2.97	−1.87	−2.40	1.30	1.22
	[13y–17]	24	−2.62	−3.28	−1.96	−2.23	1.57	2.27
	≥18y	24	−1.90	−2.31	−1.48	−1.84	0.99	1.25
AI	[8y–10y]	24	−0.40	−1.14	0.35	−0.36	1.76	2.02
	[10y–11y6m]	24	−0.70	−1.48	0.07	−0.57	1.85	2.15
	[11y6m–13y]	24	−0.55	−1.07	−0.03	−0.59	1.22	1.58
	[13y–17]	24	−0.97	−1.45	−0.50	−0.86	1.12	0.92
	≥18y	24	−1.08	−1.60	−0.56	−0.77	1.23	1.58

**Table 3:** Average equivalent attenuation (EA) and asymmetry index (AI) values for male and female patients (all ages combined).

Variables	Sex	N	Mean	Lower	Upper	Median	SD	IQR
EA _right_	F	57	−3.54	−4.00	−3.07	−3.42	1.74	2.24
	M	63	−3.31	−3.77	−2.85	−2.88	1.82	2.23
EA _left_	F	57	−2.80	−3.25	−2.36	−2.58	1.68	2.30
	M	63	−2.57	−2.90	−2.23	−2.40	1.32	1.93
AI	F	57	−0.73	−1.13	−0.34	−0.71	1.48	1.47
	M	63	−0.75	−1.11	−0.38	−0.60	1.47	1.63

**Table 4:** Average equivalent attenuation (EA) and asymmetry index (AI) values for male and female patients and each age group.

Variables	Age Group	Sex	N	Mean	Lower	Upper	Median	SD	IQR
EA_right_	[8y–10y]	F	12	−4.16	−5.07	−3.24	−3.74	1.44	1.18
		M	12	−3.18	−4.62	−1.74	−2.40	2.27	1.71
	[10y–11y6m]	F	12	−3.81	−4.78	−2.84	−3.59	1.53	2.11
		M	12	−3.95	−5.14	−2.77	−3.79	1.86	3.14
	[11y6m–13y]	F	12	−3.15	−4.25	−2.04	−2.63	1.74	1.56
		M	12	−2.79	−3.68	−1.90	−2.59	1.40	2.37
	[13y–17]	F	10	−3.64	−5.24	−2.04	−2.91	2.24	2.53
		M	14	−3.56	−4.79	−2.34	−3.62	2.12	2.30
	≥18y	F	11	−2.89	−4.06	−1.72	−2.17	1.74	2.04
		M	13	−3.04	−3.82	−2.26	−3.04	1.30	1.64
EA_left_	[8y–10y]	F	12	−3.72	−4.99	−2.46	−3.69	1.98	2.71
		M	12	−2.83	−3.65	−2.00	−2.79	1.30	1.45
	[10y–11y6m]	F	12	−3.10	−4.09	−2.12	−3.48	1.55	2.30
		M	12	−3.25	−4.23	−2.27	−3.24	1.54	2.10
	[11y6m–13y]	F	12	−2.42	−3.37	−1.47	−2.43	1.49	1.22
		M	12	−2.42	−3.15	−1.69	−2.28	1.15	1.05
	[13y–17y]	F	10	−2.95	−4.24	−1.66	−2.64	1.81	2.02
		M	14	−2.39	−3.19	−1.59	−1.99	1.39	1.83
	≥18y	F	11	−1.75	−2.39	−1.12	−1.58	0.94	1.25
		M	13	−2.02	−2.65	−1.38	−1.92	1.05	0.94
AI	[8y–10y]	F	12	−0.43	−1.67	0.80	−0.83	1.94	2.53
		M	12	−0.36	−1.40	0.68	−0.02	1.64	1.68
	[10y–11y6m]	F	12	−0.71	−1.88	0.47	−0.46	1.85	2.16
		M	12	−0.70	−1.93	0.52	−0.69	1.93	2.16
	[11y6m–13y]	F	12	−0.72	−1.31	−0.14	−0.81	0.93	1.46
		M	12	−0.37	−1.32	0.57	−0.31	1.49	1.49
	[13y–17y]	F	10	−0.69	−1.29	−0.09	−0.77	0.84	0.65
		M	14	−1.17	−1.91	−0.43	−0.93	1.28	1.08
	≥18y	F	11	−1.14	−2.19	−0.08	−0.71	1.57	1.31
		M	13	−1.02	−1.58	−0.47	−0.83	0.91	1.38

## Data Availability

The data presented in this study are available on request from the corresponding author. The data are not publicly available due to ethical, legal, and privacy issues.

## References

[1] Pickles J.O. (2015). Auditory pathways: Anatomy and physiology. Handb. Clin. Neurol..

[2] Seldana E. (2015). All the way from the cortex: A review of auditory cortico-subcollicular pathways. Cerebellum.

[3] Terreros G., Delano H.P. (2015). Corticofugal modulation of peripheral auditory responses. Front. Syst. Neurosci..

[4] Winer J.A. (2005). Decoding of the auditory corticofugal systems. Hear. Res..

[5] Warr W.B., Guinan J.J., White J.S., Altshuler R.A., Hoffman D.W., Bobbin R.P. (1986). Organization of efferent fibers: The lateral and medial olivocochlear systems. Neurobiology of Hearing: The Cochlea.

[6] Lopez-Poveda E.A. (2018). Olivocochlear efferents in animals and humans: From anatomy to clinical relevance. Front. Neurol..

[7] Bajo V.M., Moore D.R. (2005). Descending projections from the auditory cortex to the inferior colliculus in the gerbils, *Meriones unguiculatus*. J. Comp. Neurol..

[8] Jäger K., Kössl M. (2016). Corticofugal modulation of DPOAEs in gerbils. Hear. Res..

[9] Ponton C., Eggermont J.J., Khosla D., Kwong B., Don M. (2002). Maturation of human central auditory system activity: Separating auditory evoked potentials by dipole source modeling. Clin. Neurophysiol..

[10] Sharma A., Martin K., Rol P., Bauer P., Sweeney M.H., Gilley P., Dorman M. (2005). P1 latency as a biomarker for central auditory development in children with hearing impairment. J. Am. Acad. Audiol..

[11] Seither-Preisler A., Johnson L., Krumbholz K., Nobbe A., Patterson R., Seither S., Lütkenhöner B. (2007). Tone sequences with conflicting fundamental pitch and timbre changes are heard differently by musicians and nonmusicians. J. Exp. Psychol. Hum. Percept. Perform..

[12] Dehaene-Lambertz G., Spelke E.S. (2015). The Infancy of the Human Brain. Neuron.

[13] Hall J.W. (2007). New Handbook of Auditory Evoked Responses.

[14] Spitzer E., White-Schwoch T., Woodruff Carr K., Skoe E., Kraus N. (2015). Continued maturation of the click-evoked auditory brainstem response in preschoolers. J. Am. Acad. Audiol..

[15] Krizman J., Tierney A., Fitzroy A.B., Skoe E., Amar J., Kraus N. (2015). Continued maturation of auditory brainstem function during adolescence: A longitudinal approach. Clin. Neurophysiol..

[16] Eggermont J.J. (2014). Development of Central Auditory Nervous System. Handbook of Central Auditory Processing Disorder.

[17] Moore J.K., Linthicum F.H. (2007). The human auditory system: A timeline of development. Int. J. Audiol..

[18] Elliott L.L. (1979). Performance of children aged 9 to 17 years on a test of speech intelligibility in noise using sentence material with controlled word predictability. J. Acoust. Soc. Am..

[19] Hensch T.K. (2005). Critical period plasticity in local circuits. Nat. Rev. Neurosci..

[20] Kemp D.T. (1978). Stimulated otoacoustic emissions from within the human auditory system. J. Acoust. Soc. Am..

[21] Kemp D.T. (2002). Otoacoustic emissions, their origin in cochlear function, and use. Br. Med. Bull..

[22] Collet L., Kemp D.T., Veuillet E., Duclaux R., Moulin A., Morgon A. (1990). Effect of contralateral auditory stimuli on active cochlear micromechanical properties in human subjects. Hear. Res..

[23] Veuillet E., Collet L., Duclaux R. (1991). Effect of contralateral acoustic stimulation on active cochlear micromechanical properties in human subjects: Dependence on stimulus variables. J. Neurophysiol..

[24] Dragicevic C.D., Aedo C., Leon A., Bowen M., Jara N., Terreros G., Robles L., Delano P.H. (2015). The olivocochlear reflex strength and cochlear sensitivity are independently modulated by auditory cortex microstimulation. J. Assoc. Res. Otolaryngol..

[25] Aedo C., Terreros G., Leon A., Delano P.H. (2016). The corticofugal effects of auditory cortex microstimulaton on auditory nerve and superior olivary complex responses are mediated via alpha-9 nicotinic receptor subunit. PLoS ONE.

[26] Schofield B.R. (2010). Structural organization of the descending auditory pathway. The Oxford Handbook of Auditory Science: The Auditory Brain.

[27] Suthakar K., Ryugo D.K. (2017). Descending projections from the inferior colliculus to medial olivocochlear efferents: Mice with normal hearing, early onset hearing loss, and congenital deafness. Hear. Res..

[28] Khalfa S., Veuillet E., Collet L. (1998). Influence of handedness on peripheral auditory asymmetry. Eur. J. Neurosci..

[29] Bidelman G.M., Bhagat S.P. (2015). Right-ear advantage drives the link between olivocochlear efferent “antimasking” and speech-in-noise listening benefits. Neuroreport.

[30] Veuillet E., Georgieff N., Philibert B., Dallery J., Marie-Cardine M., Collet L. (2001). Abnormal peripheral auditory asymmetry in schizophrenia. J. Neurol. Neurosurg. Psychiatry.

[31] Khalfa S., Bougeard R., Morand N., Veuillet E., Isnard J., Guenot M., Ryvlin P., Fischer C., Collet L. (2001). Evidence of peripheral auditory activity modulation by the auditory cortex in humans. Neuroscience.

[32] Perrot X., Ryvlin P., Isnard J., Guénot M., Catenoix H., Fischer C., Mauguière F., Collet L. (2006). Evidence for corticofugal modulation of peripheral auditory activity in humans. Cereb. Cortex.

[33] Yakunina N., Tae W.S., Kim S.S., Nam E.C. (2019). Functional MRI evidence of the cortico-olivary efferent pathway during active auditory target processing in humans. Hear. Res..

[34] Morlet T., Hamburger A., Kuint J., Ari-Even Roth D., Gartner M., Muchnik C., Collet L., Hildesheimer M. (2004). Assessement of medial olivocochlear system function in pre-term and fullterm newborns using a rapid test of transient optoacoustic emissions. Clin. Otolaryngol..

[35] Carvallo R.M.M., Sanches S.G.G., Ibidi S.M., Soares J.C., Durante A.S. (2015). Efferent inhibition of optoacoustic emissions in preterm neonates. Braz. J. Otorhinolaryngol..

[36] Morlet T., Goforth L., Hood L.J., Ferber C., Duclaux R., Berlin C.I. (1999). Development of human cochlear active mechanism asymmetry: Involvement of the medial olivocochlear system?. Hear. Res..

[37] Gkoritsa E., Tsakanikos M., Korres S., Dellagrammaticas H., Apostolopoulos N., Ferekidis E. (2006). Transient otoacoustic emissions in the detection of olivocochlear bundle maturation. Int. J. Pediatr. Otorhinolaryngol..

[38] Chabert R., Guitton M.J., Amram D., Uziel A., Pujol R., Lallemant J.G., Puel J.L. (2006). Early maturation of evoked optoacoustic emissions and medial olivocochlear reflex in preterm neonates. Pediatr. Res..

[39] Jedrzejczak W.W., Pilka E., Skarzynski P.H., Skarzynski H. (2020). Contralateral suppression of otoacoustic emissions in pre-school children. Int. J. Pediatr. Otorhinolaryngol..

[40] Clarke E.M., Ahmmed A., Parker D., Adams C. (2006). Contralateral suppression of otoacoustic emissions in children with specific language impairment. Ear Hear..

[41] Sanchez S.G.G., Carvallo R.M. (2006). Contralateral suppression of transient evoked otoacoustic emissions in children with auditory processing disorder. Audiol. Neurotol..

[42] Veuillet E., Magnan A., Ecalle J., Thai-Van H., Collet L. (2007). Auditory processing disorder in children with reading disabilities: Effect of audiovisual training. Brain.

[43] Akbari M., Panahi R., Valadbeigi A., Nahrani M.H. (2020). Speech-in-noise perception ability can be related to auditory efferent pathway function: A comparative study in reading impaired and normal reading children. Braz. J. Otorhinolaryngol..

[44] Burguetti F.A.R., Carvallo R.M.M. (2008). Efferent auditory system: Its effect on auditory processing. Braz. J. Otorhinolaryngol..

[45] Yalçinkaya F., Yilmaz S.T., Muluk N.B. (2010). Transient evoked otoacoustic emissions and contralateral suppressions in children with auditory listening problems. Auris Nasus Larynx.

[46] Angeli M.L.d.S.A., De Almeida C.I.R., Sens P.M. (2008). Comparative study between school performance on first grade children and suppression of otoacoustic transient emission. Braz. J. Otorhinolaryngol..

[47] Oldfield R.C. (1971). The assessment and analysis of handedness: The Edinburgh inventory. Neuropsychologia.

[48] Lefavrais P. (1965). Test de l’Alouette.

[49] Garinis A.C., Glattke T., Cone-Wesson B.K. (2008). TEOAE suppression in adults with learning disabilities. Int. J. Audiol..

[50] Ryan S., Kemp D.T. (1996). The influence of evoking stimulus level on the neural suppression of transient evoked otoacoustic emissions. Hear. Res..

[51] Veuillet E., Bazin F., Collet L. (1999). Objective evidence of peripheral auditory disorders in learning-impaired children. J. Audiol. Med..

[52] O-Uchi T., Kanzaki J., Satoh Y., Yoshihara S., Ogata A., Inoue Y., Mashino H. (1994). Age-related changes in evoked otoacoustic emission in normal-hearing ears. Acta Otolaryngol. Suppl..

[53] Collet L., Moulin A., Gartner M., Morgon A. (1990). Age-related changes in evoked otoacoustic emissions. Ann. Otol. Rhinol. Laryngol..

[54] Moore J.K., Guan Y.L. (2001). Cytoarchitectural and axonal maturation in human auditory cortex. J. Assoc. Res. Otolaryngol..

[55] Moncrieff D.W. (2011). Dichotic listening in children: Age-related changes in direction and magnitude of ear advantage. Brain Cogn..

[56] Jerger J., Martin J. (2004). Hemispheric asymmetry of the right ear advantage in dichotic listening. Hear. Res..

[57] Mcfadden D., Martin G.K., Stagner B.B., Maloney M.M. (2009). Sex differences in distortion-product and transient-evoked otoacoustic emissions compared. J. Acoust. Soc. Am..

[58] Abdollahi F.Z., Lotfi Y. (2011). Gender difference in TEOAEs and contralateral suppression of TEOAEs in normal hearing adults. Iran Rehabil. J..

[59] Nisha K.V., Loganathan M.K., Prabhu P. (2023). Gender differences in contralateral suppression of spontaneous otoacoustic emissions in individuals with auditory neuropathy spectrum disorders. Eur. Arch. Otorhinolaryngol..

[60] Stuart A., Cobb K.M. (2015). Reliability of measures of transient evoked otoacoustic emissions with contralateral suppression. J. Commun. Disord..

[61] Jedrzejczak W.W., Pilka E., Pastucha M., Kochanek K., Skarzynski H. (2022). The reliability of contralateral suppression of otoacoustic emissions is greater in women than in men. Audiol. Res..

